# Calorie restriction increases the sensitivity of progeroid *Ercc1*^*Δ/*−^ mice to acute (neuro)inflammation

**DOI:** 10.1007/s11357-024-01347-1

**Published:** 2024-09-17

**Authors:** V. A. Reitsema, L. Schreuder, E. Gerrits, B. J. L. Eggen, M. Goris, J. D. Laman, S. E. de Rooij, E. M. Wesseling, H. R. Bouma, R. H. Henning

**Affiliations:** 1 Department of Clinical Pharmacy and Pharmacology, University Medical Center Groningen, University of Groningen, Groningen, The Netherlands; 2 Department of Internal Medicine, University Center for Geriatric Medicine, University Medical Center Groningen, University of Groningen, Groningen, The Netherlands; 3 Department of Biomedical Sciences of Cells & Systems, Section Molecular Neurobiology, University Medical Center Groningen, University of Groningen, Groningen, The Netherlands; 4 Department of Internal Medicine, University Medical Center Groningen, University of Groningen, Groningen, The Netherlands; 5 Department of Acute Care, University Medical Center Groningen, University of Groningen, Groningen, The Netherlands; 6 Department of Pathology and Medical Biology, University Medical Center Groningen, University of Groningen, Groningen, The Netherlands

**Keywords:** Delirium, Calorie restriction, Fasting, Sepsis, Encephalopathy

## Abstract

Hospitalized elderly patients frequently suffer from delirium, especially in the context of sepsis-associated encephalopathy. Current treatments of delirium are merely symptomatic. Calorie restriction (CR) is both a promising strategy to protect against sepsis and has beneficial effects on aging-induced neurodegeneration. In this study, we investigated whether six weeks of 30% CR had protective effects on lipopolysaccharide (LPS) induced (neuro)inflammation in wild-type (WT) and progeroid mice deficient in the DNA excision-repair gene *Ercc1* (*Ercc1*^*Δ*/−^). While CR did not affect the LPS-induced inflammatory response in WT mice, CR exaggerated the peripheral inflammatory response in *Ercc1*^*Δ/*−^ mice, as evidenced by an increase of pro-inflammatory serum cytokines (TNF-α, IL-1β, and IFN-γ) and kidney injury marker Ngal. Neuroinflammatory effects were assessed by RNA-sequencing of isolated microglia. Similarly, CR did not affect microglia gene expression in WT mice, but increased neuroinflammation-associated gene expression in *Ercc1*^*Δ/*−^ mice. In conclusion, CR increases the peripheral and brain inflammatory response of *Ercc1*^*Δ/*−^ mice to a systemic inflammatory stimulus.

**Supplementary Information:**

The online version contains supplementary material available at 10.1007/s11357-024-01347-1.

## Introduction

Delirium is an acute confusional state that is associated with increased morbidity, prolonged hospital stay, and mortality and is a common complication of infection among hospitalized elderly patients. The incidence of delirium ranges from 10% (delirium present at presentation in the emergency department) to over 75% among patients admitted to the intensive care unit [1]. Hallmarks of delirium include an acute disturbance of attention and awareness. Surgery, acute (exacerbation of) illnesses, and infections are the most common precipitating factors for the development of delirium. Delirium in sepsis depends on a secondary activation of microglia in response to systemic inflammation, as shown both in animal studies [2] and in post-mortem studies of humans [3, 4]. Predisposing factors for delirium include cognitive and functional disabilities, earlier episodes of delirium, and old age. Delirium is associated with increased length of hospital stay, institutionalization rates, as well as increased conversion into cognitive dysfunction, and higher institutionalization and mortality rates [5, 6]. The pathophysiology is thought to be multifactorial, involving neurotransmitter imbalance and neuroinflammation [1].

Research on delirium in hospitalized elderly patients is hampered by the lack of suitable models that exhibit neuroinflammation in a neurodegenerative aging setting. Adult mice only show minor features of spontaneous neurodegeneration, whereas studying naturally aged mice is time-consuming, laborious, and expensive. DNA damage is a major factor contributing to aging [7, 8]. Therefore, we chose a mouse model displaying progressive severe neurodegeneration in the context of premature aging. The *Ercc1*^*Δ/*−^ mouse, deficient in the DNA excision-repair gene *Ercc1*, is a valid model of accelerated aging with a median lifespan of 10–13 weeks [7, 9], and has excessive microglia inflammatory cytokine production after a peripheral inflammatory stimulus with low-dose lipopolysaccharide (LPS) [10]. *Ercc1*^*Δ/*−^ mice lack one functional *Ercc1* allele, while the ∆ allele is a hypomorph, encoding a 7 amino-acid C-terminally truncated ERCC1 protein with lower binding to the Xpf subunit of the Ercc1/Xpf heterodimeric structure-specific endonuclease complex and therefore has strongly reduced stability compared to the WT protein [11–14]. The *Ercc1*^*Δ/*−^ mouse model resembles several aspects predisposing for delirium in frail elderly, as it shows signs of accelerated aging, neurodegeneration, multimorbidity, and priming of microglia [10, 14–16]. We chose to focus on microglia in our study because these cells are the primary immune responders in the central nervous system, playing a crucial role in neuroinflammation and age-related neurodegenerative processes.

Calorie restriction (CR) has beneficial effects on age-related neuroinflammation in wild-type mice [17, 18] and reduces neurodegenerative pathology, astrogliosis, and microgliosis in *Ercc1*^*Δ*/−^ mice while extending life span [9]. Previously, we showed that a low-fat diet combined with CR prevents the activation of microglia in aged mice compared to a high-fat diet [19]. In the presence of a peripheral inflammatory stimulus, a brief period of moderate CR protects against sepsis-induced morbidity and mortality [20, 21] while longer and more severe CR increases mortality [22, 23]. Furthermore, CR suppresses the brain inflammatory response to a peripheral inflammatory stimulus in adult rats [24]. These studies investigated the role of CR on age-related neuro-inflammation or (neuro)inflammation in response to a peripheral inflammatory stimulus. Yet, the effect of CR on the acute neuro-inflammatory response to a peripheral inflammatory trigger in aged mice is unknown. Hence, we addressed whether moderate CR can protect progeroid (*Ercc1*^*Δ/*−^) mice from LPS-induced (neuro)inflammation. To this end, we quantified the peripheral inflammatory response by measuring circulating cytokines and expression of inflammatory markers in the kidney, which we related with microglia gene expression after LPS challenge of ad libitum fed and CR WT and *Ercc1*^*Δ/*−^ mice.

## Methods

### Mice

All experiments were approved by the Institutional Animal Care and Use Committee of the University Medical Center Groningen (IvD 15167–01), in accordance with Dutch animal care and use laws. The generation and characterization of *Ercc1*^*Δ/*−^ mice was previously described [14]. In brief, *Ercc1*^*Δ/*+^ and *Ercc1*^+*/*−^ (on a FVB or C57/BL6 background, respectively) mice were crossed to yield *Ercc1*^*Δ/*−^ mice mutants in a uniform C57Bl6J/FVB F1 hybrid background. As controls, littermates carrying wild-type *Ercc1* allele(s) were used. Both males and females were included. Offspring were genotyped by PCR after weaning using primers listed in Table 1. Mice were individually housed in conventional cages and weight was monitored weekly till 10 weeks of age and twice per week afterwards.

**Table 1:** Primers for genotyping

Allele	Product size	Forward primer 5′-3′	Reverse primer 5′-3′
WT	246 bp	AGCCGACCTCCTTATGGAAA	ACAGATGCTGAGGGCAGACT
KO	390 bp	TCGCCTTCTTGACGAGTTCT	ACAGATGCTGAGGGCAGACT
292 (Δ)	530 bp	TCGCCTTCTTGACGAGTTCT	CTAGGTGGCAGCAGGTCATC

### Food composition and intake

All mice were bred and fed an AIN93G diet (Research Diet Services B.V.; gross energy content 4.9 kcal/g dry mass, and digestible energy of 3.97 kcal/g). Food intake was monitored daily. Mice were fed just before the start of the dark (active) period, corresponding to their usual eating time, to avoid changes in circadian rhythm. WT and *Ercc1*^*Δ/*−^ mice consumed on average 3.0 g and 2.3 g of food per day when fed a calorie neutral diet to maintain a stable body weight (ad libitum). CR was initiated relative to the normal intake of food when animals were 9 weeks of age, with a gradual restriction of 10% per week until 30% CR after 2 weeks, resulting in an average food consumption of WT and *Ercc1*^*Δ/*−^ mice of 2.1 g and 1.6 g/day respectively. CR mice were kept at 30% CR for another 4 weeks (a total of 6 weeks CR).

### Temperature logger implantation

Prior to initiation of CR a temperature logger (DST nano-T, Starr Oddi) was surgically implanted in the intraperitoneal cavity under sterile conditions at 7 weeks of age. Mice were anesthetized under isoflurane anesthesia (induction: 4% isoflurane, flowrate 0.6–0.8 L/min; maintenance 2.5% isoflurane, flow rate 0.4 L/min) followed by a midline abdominal incision, opening of the peritoneum and implantation of the temperature logger, a procedure taking about 10 min. Subsequently, carprofen (5 mg/kg) was administered subcutaneously as analgesic. Mice were allowed to recover for 1 week before being subjected to CR.

### Induction of LPS challenge

After 6 weeks of CR, at the age of 15 weeks, mice were injected i.p. with LPS at 1 mg/kg body weight (Sigma-Aldrich, *Escherichia coli*, 0111:B4) diluted in PBS to 1 μL/g body weight. Sham controls were injected i.p. with a similar volume of PBS. Three hours following injection mice were terminated under deep anesthesia (4% isoflurane with 7.5% O_2_ in air) by exsanguination via cardiac puncture. Subsequently, mice were transcardially perfused with PBS. Blood was collected in EDTA anticoagulant or allowed to clot. Kidneys were harvested, flash frozen in liquid nitrogen and kept at – 80 °C until further analysis. Brains were removed and kept in cold medium A (HBSS [Gibco] with 0.6% glucose [Sigma] and 7.5 mM HEPES [Lonza]) until further processing.

### Luminex assay

Serum was collected from clotted blood by centrifugation for 10 min at 3000 × g, and stored at – 80 °C until analysis by Luminex assay for IL-6, TNF-α, IL-1β, IL-33, IFN-γ, IL-12p70, and IL-10. Samples were diluted two-fold with PBS and measured by Luminex using R&D systems magnetic Luminex assay kit using a standard protocol. In brief, 50 µL of standard or sample was added to each well followed by addition of 50 µL of microparticle cocktail and incubated at an orbital microplate shaker (800 rpm) for 2 h. Plates were then washed with wash buffer using a magnetic device. Subsequently, 50 µL of biotin-antibody cocktail was added and incubated at RT for 1 h on the shaker (800 rpm), and immediately washed with PBS. Then, wells were incubated with 50 µL of streptavidin-PE for 30 min on the shaker. After washing, the optical density was read using a Luminex 200.

### RNA isolation

Kidney RNA was isolated according to the manufacturer’s instructions of Nucleospin II (Macherey–Nagel). Total RNA concentration was measured with a NanoDrop2000 spectrophotometer (Thermo Scientific).

### Quantitative real-time-PCR

Reverse transcription was performed with random hexamers, recombinant RNasin ribonuclease inhibitor, and Moloney murine leukemia virus reverse transcriptase and dNTP and RT Buffer (all from Promega). Quantitative PCR reactions were performed using SYBR Green supermix with ROX (Bio-Rad) in an ABI 7900HT real-time thermal cycler. For each gene, measurements were performed in duplicate. PCR primers were designed in Primer-BLAST software 40. Primer sequences are provided in Table 2.

**Table 2:** Primers for quantitative RT-PCR

Gene	Forward primer 5′-3′	Reverse primer 5′-3′
*Gapdh*	GCAAATTCAACGGCACAG	CACCAGTAGACTCCACGAC
*Icam1*	GCCCTGCAATGGCTTCAACC	TGGCGGCTCAGTATCTCCTC3
*Ngal*	ACGGACTACAACCAGTTCGC	AATGCATTGGTCGGTGGGG
*Il6*	GAGGATACCACTCCCAACAGACC	AAGTGCATCATCGTTGTTCATACA3
*Tnfα*	TCTTCTGTCTACTGAACTTCGG	AAGATGATCTGAGTGTGAGGG
*Il1β*	CCCAAAAGATGAAGGGCTGC	TGATACTGCCTGCCTGAAGC

### Isolation of microglia and cell sorting

Microglia were isolated as described in detail previously (Galatro et al., 2017). Briefly, brain tissue was mechanically homogenized in cold medium A, followed by centrifugation at 220 RCF, 4 °C for 10 min. The pellet was resuspended in 22% Percoll (GE Healthcare) in myelin gradient buffer (5.6 mM NaH_2_PO_4_.2H_2_O, 20 mM Na_2_HPO_4_.2H_2_O, 140 mM NaCl, 5.4 mM KCl, 11 mM glucose), and centrifuged for 20 min at 950 RCF at 4 °C to remove the myelin. Finally, the cell pellet was incubated with phycoerythrin (PE)-coupled rat anti-mouse CD11b (Clone M1/70, eBioscience), FITC-coupled rat anti-mouse CD45 (Clone 30-F11, eBioscience), APC-coupled rat anti-mouse Ly6c (Clone HK 1.4, Biolegend). In order to identify single cells, forward and side scatter parameters were used, and live cells were selected via the exclusion of DAPI-negative cells. Microglia were sorted by gating CD11b^pos^/CD45^int^/Ly6c^neg^/DAPI^neg^ cells on a Beckman Coulter MoFlo Astrios or XDP. A representative FACS plot is shown in Supplemental Fig. 1.

### Library Quantseq 3′ mRNA-seq preparation

Total RNA was isolated from FACS-sorted microglia using the QIAGEN RNA isolation kit according to the manufacturer’s instructions. RNA quantity and quality were analyzed on a fragment analyzer. Sequencing libraries were prepared with the Quantseq 3′ mRNA-Seq Library Prep Kit FWD. RNA sequencing was conducted using Lexogen’s 3′ QuantSeq method, utilizing single-end sequencing with a read depth ranging from 2.4 million to 6 million reads per sample.

### RNA-sequencing analysis

Prior to alignment, quality control of the raw FASTQ files was performed with FASTQC. Bad quality bases were trimmed with TrimGalore version 0.4.5. Sequences were aligned using HiSat2 version 2.1 to the *Mus musculus* (GRCm38.91) reference template obtained from Ensembl and quantified with featureCounts. A quality check of aligned data was done with FASTQC and MultiQC and showed high-quality alignment of all the samples (75–85%). Raw count matrices were loaded in R (v4.0.4) and annotated by converting the ensemble IDs to gene symbols using the corresponding gtf file. Only genes with > 1 counts in at least 2 samples were included in the analysis. Principal component analysis (PCA) on the count matrix was performed on VST transformed counts using DESeq2 (v1.30.1) [25]. Differential gene expression analysis was performed with the edgeR package from Bioconductor (v3.32.1) [26]. Differential gene expression analysis was performed using an absolute log fold change > 1 and a FDR-adjusted *P* value < 0.05 as cutoffs. All differentially expressed genes are listed in supplementary Table 1. Gene ontology (GO) enrichment was performed with MetaScape [27].

### Statistical analysis

Results are expressed as mean ± standard error of the mean. Data were analyzed using R and figures created with GraphPad Prism 5 software (GraphPad Software, Inc.). Figures of RNA-sequencing data were generated by R 4.0.4. in Rstudio 1.1.383. Comparisons of two groups were analyzed by two-sided *T* test and multiple groups by two-way analysis of variance (ANOVA) followed by post hoc analysis using Bonferroni’s multiple-comparison test. A *P* value < 0.05 was considered statistically significant.

## Results

### Moderate calorie restriction reduces body weight

**Fig. 1:**
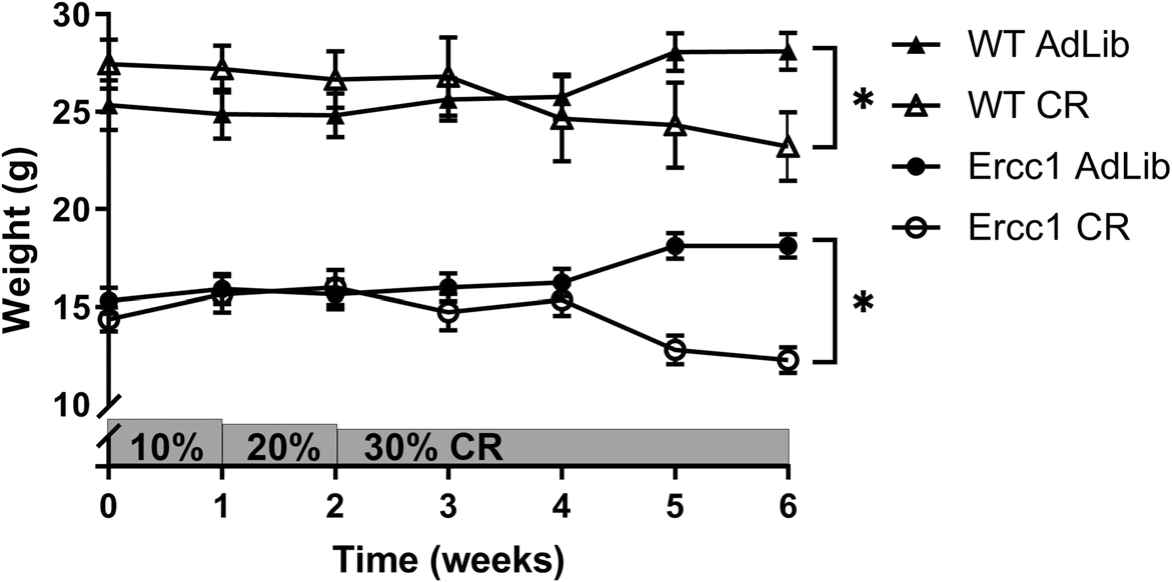
Calorie restriction reduces body weight more severely in *Erccl*^*Δ/*^.^−^ mice in comparison to wild-type mice. Comparison of body weight at baseline and during CR, which started at the age of 9 weeks. Body weights between AdLib and CR animals (within genotype) were compared by a two-sided *T* test and different at weeks 5 and 6. Bars represent mean weight ± SEM. *P < 0.05

To determine the effect of CR, body weight was closely monitored throughout the study. Moderate CR resulted in a reduction of body weight of WT mice by 17% (± 5.0%) compared to ad libitum fed WT mice (Fig. 1). As expected, the starting weight of *Erccl*^*Δ/−*^ mice was lower than WT mice (15 ± 1.7 g versus 28 ± 4.8 g respectively). Moderate CR reduced the body weight of *Erccl*^*Δ/−*^ mice with 32% (± 8.5%).

### Validation of the induction of a peripheral inflammatory response by LPS

To confirm the efficacy of i.p. injection of 1 mg/kg LPS to induce an inflammatory response, we measured the peripheral inflammatory response in ad libitum fed WT mice after injection of LPS in comparison to PBS. LPS was injected after 6 weeks of CR, at the age of 15 weeks. LPS-challenge resulted in the induction of a peripheral inflammatory response, as shown by a rise in body temperature (Supplemental Fig. 2A) and increased serum levels of the pro-inflammatory cytokines IL-6, TNF-α, IL-1β, immunoregulatory cytokine IL-12p70, and anti-inflammatory cytokine IL-10 (Supplemental Fig. 2B–H). Additionally, LPS-injection resulted in upregulation of genes encoding for intercellular adhesion molecule (*Icam1*) and the early marker of acute kidney injury neutrophil gelatinase-associated lipocalin (*Ngal*) in the kidney (Supplemental Fig. 2I and J). Thus, injection of LPS effectively induced a peripheral pro-inflammatory response demonstrated by increased inflammatory cytokine levels in serum, associated with upregulation of markers for acute kidney injury.

### The peripheral inflammatory response is augmented by moderate CR in Ercc1^Δ/−^ mice

**Fig. 2:**
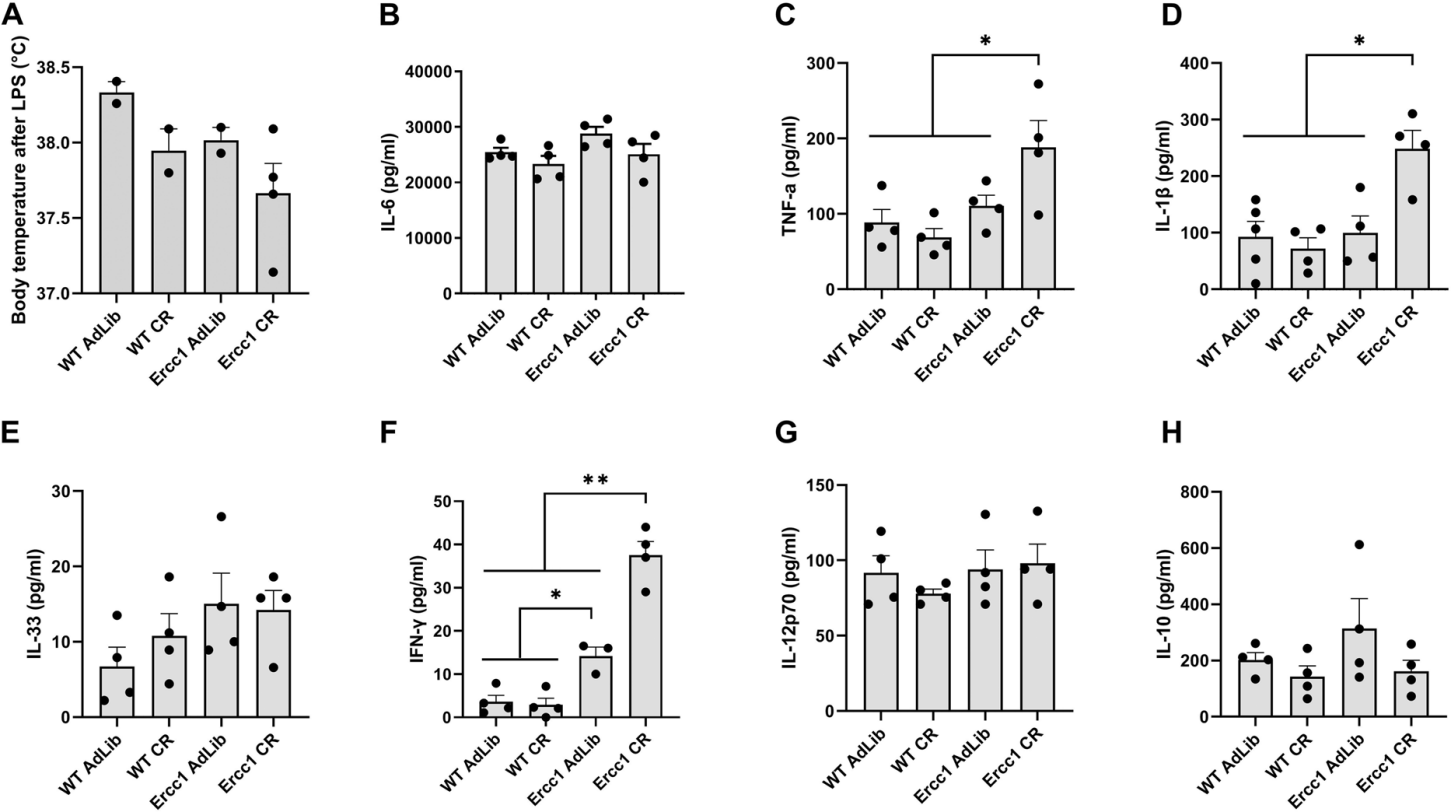
Calorie restriction increases the LPS-induced peripheral inflammatory response in *Erccl*^*Δ/*^.^−^ mice in comparison to wild-type mice. **A** Body temperature before and 3 h after LPS-injection at the age of 15 weeks. **B**–**H** Serum cytokines measured by Luminex assay. Bars represent mean ± SEM. Statistical analysis by two-way ANOVA and post-hoc Bonferroni. *P < 0.05; **P < 0.01; *ns* non-significant; sample size *n* = 3–5 per group (except for body temperature, dots represent individual animals)

Next, we assessed the effect of CR on peripheral inflammation after LPS-injection in WT and *Erccl*^*Δ/−*^ mice. In LPS-challenged WT mice, moderate CR did not affect the peripheral inflammatory response, as neither body temperature (Fig. 2A), nor levels of serum cytokines (Fig. 2B–H) in CR mice were different from those observed after LPS challenge in WT mice fed ad libitum. The deficiency of *Ercc1* in the context of ad libitum feeding did not affect the systemic inflammatory response to LPS compared to WT mice. No differences were found in serum cytokine levels between groups, except for an increase in the level of IFN-γ after LPS challenge in both ad libitum fed and CR *Erccl*^*Δ/−*^ mice after LPS injection, as compared to both groups of WT mice (Fig. 2G). In contrast, CR in *Erccl*^*Δ/−*^ mice increased cytokine levels (TNF-α, IL-1β, and a further increase in IFN-γ) in response to LPS, as compared to all other groups.

In LPS-challenged WT mice, markers of kidney inflammation were mostly unaffected by CR (Supplemental Fig. 3A–D), except for an increase of endothelial activation marker *Icam1* (Supplemental Fig. 3E). Deficiency of *Ercc1* alone did not affect kidney injury or inflammation in comparison to WT mice after LPS injection, yet CR in *Erccl*^*Δ/−*^ mice upregulated markers for acute kidney injury and inflammation (*Ngal*, *Il6*, and *Tnfα*) (Supplemental Fig. 3). Thus, while CR nor deficiency of *Ercc1* affected the peripheral inflammatory response to LPS, CR in *Erccl*^*Δ/−*^ mice led to an augmented peripheral inflammatory response, involving an upregulation of kidney inflammatory and damage markers.

### The microglial inflammatory response to LPS is exaggerated in Ercc1^Δ/−^ mice

We initially validated whether i.p. injection of LPS elicited a neuroinflammatory response, secondary to peripheral inflammation, by performing RNA-sequencing on isolated microglia and comparing inflammation-related genes in WT mice injected with either PBS or LPS (“LPS-enriched genes”). In WT mice, LPS induced an upregulation of inflammation-related genes in comparison to PBS-injection when mice were fed ad libitum, and this upregulation was less pronounced in CR WT mice (Supplemental Fig. 4).

**Fig. 3:**
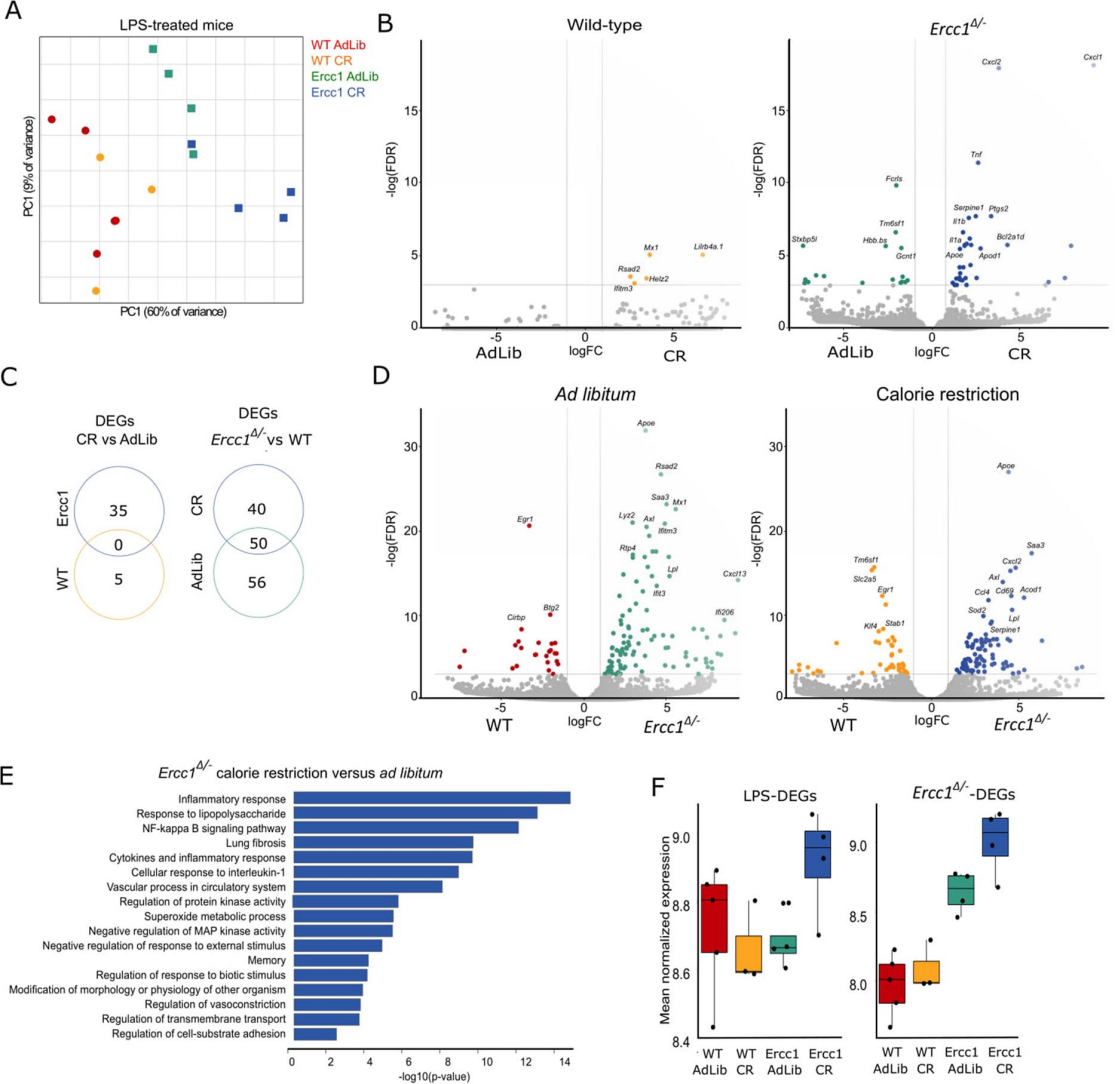
Microglia from LPS-treated *Erccl*^*Δ/*−^ mice at the age of 15 weeks exhibit an increased pro-inflammatory neurodegenerative phenotype after 6 weeks of calorie restriction. **A** Principal component analysis depicting the segregation between WT versus *Erccl*^*Δ/*−^ mice (PC1) and ad libitum fed vs. CR mice (PC2) injected with 1 mg/kg LPS i.p. **B** Volcano plots depicting calorie restriction associated DEGs in LPS injected wild type and *Erccl*^*Δ/*−^ mice. **C** Venn diagrams depicting DEGs (logFC > 1 and adjusted-*p* < 0.05) in LPS injected mice from left: ad libitum fed vs. CR WT mice (yellow) and ad libitum fed vs. CR *Erccl*^*Δ/*−^ mice (blue) and right: CR WT mice vs CR *Erccl*^*Δ/*−^ mice (blue) and ad libitum fed WT mice vs. ad libitum fed *Erccl*^*Δ/*−^*mice* (green). **D** Volcano plots depicting *Ercc1*^−*/*−^ associated DEGs in ad libitum fed and CR mice injected with LPS. **E** Bar plot depicting gene ontology terms associated with enriched genes in LPS injected ad libitum fed vs. CR *Erccl*^*Δ/*−^ mice. **F** Box plot depicting increased mean normalized expression of “LPS-enriched” genes (left panel labeled “LPS-DEGs,” see Supplementary Fig. 1) and “ERCC1-enriched” genes (right panel labeled “*Erccl*^*Δ/*−^ -DEGs”) in CR + *Erccl*^*Δ/*^.^−^ mice injected with LPS; sample size *n* = 3–5 per group (dots represent individual animals)

Next, using RNA-sequencing of isolated microglia, we investigated the effects of CR on brain inflammation upon LPS challenge from brains of LPS-treated WT and *Erccl*^*Δ/−*^ mice fed ad libitum or CR. Using PCA, a clear segregation between WT and *Erccl*^*Δ/−*^ mice was observed in the first PC, and a moderate segregation between ad libitum fed and CR mice in the second PC (Fig. 3A). Differential gene expression analysis between ad libitum fed and CR groups identified only 5 CR-associated DEGs in LPS-injected WT mice and these were all involved in interferon (IFN) mediated inflammation (*Ifitm3*, *Helz2*, *Mx1*, *Rsad2*), reflecting an increase in IFN-associated signalling after CR (Fig. 3B and C). In LPS injected *Ercc1*-deficient mice, 35 DEGs were identified between ad libitum fed and CR, indicating that the effect of CR is more pronounced in *Ercc1*^*Δ/−*^ mice than in WT mice (Fig. 3B and C). These genes included the acute phase response gene *Serpine1*, prostaglandin production (*Ptgs2*), pro-inflammatory cytokines (*Il1a*, *Il1B*, *Tnf*), and chemokines (*Cxcl1*, *Cxcl2*) (Fig. 3D). Biological processes associated with the CR-enriched genes in *Erccl*^*Δ/−*^ mice were involved in the inflammatory response, including IFN-mediated inflammation (Fig. 3D and E). While the expression of LPS-enriched genes in isolated microglia was not affected by CR in WT mice or by deficiency of *Ercc1* alone, CR in *Erccl*^*Δ/−*^ mice increased expression of LPS-enriched genes (Fig. 3F, left panel). Taken together, these findings indicate that CR enhances susceptibility of *Erccl*^*Δ/−*^ mice to microglia activation after LPS treatment.

Finally, to establish the role of CR in the vulnerability to DNA damage, we identified DEGs between PBS-injected ad libitum fed *Erccl*^*Δ/−*^ mice and WT mice to define “*Ercc1*-enriched genes.” Next, we examined this set of genes in the four LPS-treated groups (Fig. 3F, right panel). In line with our findings of LPS-enriched genes, the expression of *Ercc1*-enriched genes was not affected by CR in WT mice or by deficiency of *Ercc1* alone in LPS-treated animals. Yet, LPS treatment of CR *Erccl*^*Δ/−*^ mice most prominently enriched the set of *Ercc1*-enriched genes. These data thus indicate that both the response to an inflammatory stimulus such as LPS as well as the vulnerability to DNA damage and aging are enhanced when septic *Erccl*^*Δ/−*^ mice are moderately calorie restricted.

## Discussion

In this study, we explored the effects of caloric restriction (CR) on neuroinflammation triggered by systemic inflammation in young wild-type and ERCC1 mice as a model for delirium in elderly patients with infections. The low dose of LPS used successfully induced peripheral inflammation, as shown by elevated circulating cytokines and increased markers of kidney injury and inflammation, confirming the validity of our model for studying delirium in this context. We show that moderate CR increases the LPS-induced (neuro)inflammatory response in *Erccl*^*Δ/−*^ mice, evidenced by an increased release of cytokines, an upregulation of markers for acute kidney injury and a pro-inflammatory microglia profile, in comparison to ad libitum fed *Erccl*^*Δ/−*^ mice and WT mice (both ad libitum fed and CR). The observations in our study are opposing the general perception that, in the absence of a peripheral inflammatory response, CR reduces neuroinflammation during aging [9, 17, 18] and, in the absence of aging, CR reduces the neuroinflammatory response to a peripheral inflammatory response [24].

### Calorie restriction did not influence the inflammatory response to LPS in wild-type mice

Previous studies have established that the relationship between CR and sepsis outcome in mice is hormetic: brief and/or moderate restriction (8 days alternate day fasting, 25–50% CR up to 4 weeks) has beneficial effects on sepsis outcome [20, 21, 28], prolonged (40% CR for 20 weeks) or severe CR (75% 7 days) increases sepsis mortality [22, 23]. It is important to note that study outcomes might be affected by differences in housing conditions, implementation of CR, age, and induction of sepsis. Although these studies showed divergent effects of CR on sepsis outcome, body weight changes were comparable (ranging from 18 to 29%). Here, after a gradual decrease of food intake to 30% CR in 3 weeks, WT mice lost 17% (± 5%) of their body weight in the next 3 weeks of CR in comparison to WT mice fed ad libitum, suggesting a moderate dietary intervention. Although this regimen may have been too mild to benefit LPS injected WT mice, the 30% CR in the current study was deliberately chosen so it could also be extended to the frail progeroid *Erccl*^*Δ/−*^ mice.

### Ercc1^Δ/−^ mice rapidly lost weight after calorie restriction

As expected, *Erccl*^*Δ/−*^ mice displayed a 47% lower body weight at the start of the study in comparison to WT mice. Previously, a range of 25–50% has been described in a comparison between *Erccl*^*Δ/−*^ and WT mice [14]. In this study, CR reduced the body weight of *Erccl*^*Δ/−*^ mice to a larger extent (32 ± 8.5%) than WT mice. The effects of CR are not only dependent on the net calorie reduction, but also on the ability of CR mice to maintain body fat [29]. Maintenance of body fat improves energy reserves and modulates the release of anti-inflammatory mediators and lipoproteins by adipose tissue, binding and inactivating bacteria. This is in line with the U-shaped relationship between body weight and mortality in septic patients: patients with extremes of body weight (BMI < 20 or > 40) have the highest risk of death, while the lowest mortality rate is seen among patients with a BMI 25–40 [30]. Besides being lighter in weight, *Erccl*^*Δ/−*^ mice display a gradual further reduction in white adipose tissue during aging, resulting in lipodystrophy [14, 31]. The amplified inflammatory response after LPS in *Erccl*^*Δ/−*^ mice may thus result from a low baseline weight and an inability to maintain weight and fat depots during CR, attenuating its anti-inflammatory effects seen when adipose tissue is maintained.

### Calorie restriction augments the systemic inflammatory response to LPS in Ercc1^Δ/−^ mice

LPS-injection induced a comparable peripheral inflammatory response in WT mice fed ad libitum and CR. Ad libitum fed *Erccl*^*Δ/−*^ mice slightly increased IFN-γ in comparison to WT mice, while CR *Erccl*^*Δ/−*^ mice showed an overall increased inflammatory response to LPS-stimulation as evidenced by an increase of TNF-α, IL-1β, and IFN-γ. IFN-γ acts at the crossroads of innate and adaptive immunity, functioning as an immunoregulator promoting phagocytic cell phagocytosis and oxidative burst, inflammatory cell death, and pro-inflammatory cytokine production [32]. IFN-γ is a key cytokine in the senescence-associated secretory phenotype that contributes to aging-induced chronic inflammation (inflamm-aging) [33, 34], and is associated with sepsis-induced immunosuppression [35]. The systemic release of IL-1, and to a lesser extent IL-6 and TNF-α, activates the hypothalamo-pituitary-adrenocortical (HPA) axis resulting in systemic release of glucocorticoids, dampening the immune response [36]. Aging is associated with hyperactivation of the HPA axis, resulting in reduced sensitivity to glucocorticoid feedback due to receptor downregulation, especially in the brain [37]. As a result, elevation of cortisone levels is prolonged, resulting in behavioral deficits. Therefore, the excess of TNF-α, IL-1β, and IFN-γ in CR *Erccl*^*Δ/−*^ mice might be setting the stage for neuroinflammation, a prolonged anti-inflammatory immune response and behavioral deficits following an inflammatory insult.

### Calorie restriction promotes the pro-inflammatory and neurodegenerative phenotype after LPS-injection in Ercc1^Δ/−^ mice

Microglia are brain-resident macrophages pivotal in immune surveillance and, as the primary source of pro-inflammatory cytokines in the brain, are important mediators of neuroinflammation [38]. Upon aging, microglia acquire a primed pro-inflammatory state, and display an exaggerated response to inflammatory stimuli [39]. The release of cytokines alters neurotransmission, eliciting neuroendocrine and behavioral effects [40]. Chronic neuroinflammation contributes to the decline of function in the aging brain and increases its vulnerability to peripheral systemic inflammation [41].

In the current study, neuroinflammation in response to LPS was not increased in ad libitum fed *Erccl*^*Δ/−*^ mice in comparison to WT mice, in contrast to earlier findings of increased LPS-induced microglial activation in *Erccl*^*Δ/−*^ mice in comparison to WT mice [10]. Mice in both studies were exposed to the same dose of LPS derived from identical *E*. *coli* and euthanized at the same timepoint (3 h after injection), but the *Erccl*^*Δ/−*^ mice in the study of Raj et al. were older (16 weeks instead of 14 weeks). The discrepancy is possibly explained by age differences, as aging dysregulates both innate and adaptive immunity, a process also known as immunosenescence [34]. Immunosenescence has previously been described in elderly *Erccl*^*Δ/−*^ mice as well as in mice with selective *Ercc1* knock out in hematopoietic cells [42, 43]. Potentially, due their younger age in comparison to the study by Raj et al. (note that 2 weeks is ± 20% of their lifetime considering a median lifespan of 13 weeks) the *Erccl*^*Δ/−*^ mice in this study might not exhibit a similar level of immunosenescence and avoid an excessive inflammatory response. Alternatively, other less well identifiable factors related to husbandry, housing and composition of the diet may be involved.

As expected, microglia transcriptomics of LPS-treated ad libitum fed *Erccl*^*Δ/−*^ mice identified an enrichment of genes associated with an age-associated pro-inflammatory phenotype in comparison to ad libitum fed WT mice. These genes included *Apoe* [44], *Apod1* [45], *Lyz2*, *Axl*, and *Lpl* [46], as well as genes involved the IFN-mediated response [47–49]. Again, CR amplified the neuroinflammatory response in *Erccl*^*Δ/−*^, but not in WT mice. Enriched genes included pro-inflammatory cytokines (*Il1a*, *Il1B*, *Tnf*) and chemokines (*Cxcl1*, involved in neutrophil chemotaxis and *Cxcl2*, involved in monocyte and neutrophil chemotaxis [50]). Thus, CR does not activate a different set of genes in *Erccl*^*Δ/−*^ mice after LPS treatment but enhances the expression of genes involved in the pro-inflammatory response. It is well known that microglia in the aged brain are in a hyper-responsive state, facilitating a switch to a pro-inflammatory response [39]. While CR in the absence of a peripheral inflammatory response reduces aging-induced neuroinflammation [9, 17, 18], this study indicates that CR in the presence of a peripheral inflammatory stimulus promotes the hyper-responsive state. Taken together, CR enhances the neuroinflammatory microglia phenotype in LPS-treated *Erccl*^*Δ/−*^ mice.

In this experiment, we studied the effect of CR in wild-type and Ercc1 mice upon LPS challenge and confirmed the effect of the LPS challenge in wild-type animals. However, a potential limitation of this study is the absence of a control group of Ercc1 mice treated with a vehicle instead of LPS, which prevents us from determining the baseline inflammatory response in these mice.

## Conclusion

In conclusion, this study investigated the effect of 6-week 30% calorie restriction on LPS-induced inflammation in wild-type and progeroid *Erccl*^*Δ/−*^ mice. This study encompasses a moderate dietary intervention, which did not affect the inflammatory response in WT mice. In contrast, calorie restriction augmented the extent of (neuro)inflammation in response to a peripheral inflammatory stimulus in *Erccl*^*Δ/−*^ mice, setting the stage for development of sepsis-associated encephalopathy and delirium often induced by a peripheral stimulus like an urinary tract infection or a surgical procedure.

## Supplementary Information

Below is the link to the electronic supplementary material.

Supplemental Fig. 1. Representative FACS plots. Isolated microglia were incubated with phycoerythrin (PE)-coupled rat anti-mouse CD11b (Clone M1/70, eBioscience), FITC-coupled rat anti-mouse CD45 (Clone 30-F11, eBioscience), APC-coupled rat anti-mouse Ly6c (Clone HK 1.4, Biolegend). In order to identify single cells, forward and side scatter parameters were used, and live cells were selected via the exclusion of DAPI-negative cells. Microglia were sorted by gating CD11b^pos^/CD45^int^/Ly6c^neg^/DAPI^neg^ cells on a Beckman Coulter MoFlo Astrios or XDP. The gating strategy included R1 and R2 to sort cells, R3 for DAPI^neg^ cells, R6 for CD11b^pos^/CD45^pos^, and R4 for Ly6c^neg^ cells. (JPG 660 KB)

Supplemental Fig. 2. LPS-injection induces a peripheral inflammatory response in wild-type mice. Effect of injection of PBS or LPS in WT mice fed ad libitum at the age of 15 weeks. A: Body temperature before and 3 h after injection; B-H: Serum cytokines.; I: Kidney expression levels of *Intercellular Adhesion Molecule 1 (Icam1*) corrected for *Gapdh*; J: Kidney expression levels of *Neutrophil gelatinase-associated lipocalin (Ngal)*corrected for *Gapdh*. Bars represent mean ± SEM. Statistical analysis by two-sided T-test. * = P < 0.05; ** = P < 0.01; *** =  < 0.001; ns = non-significant; sample size n = 4–6 per group (except for body temperature, dots represent individual animals). (JPG 766 KB)

Supplemental Fig. 3. LPS treatment in combination with calorie restriction increases kidney inflammatory markers in *Erccl*^*Δ/−*^ mice versus wild-type mice. Kidney mRNA expression of *Icam1*, *Ngal*, *Il6*, *Tnfα* and *Il1β* after LPS-injection at the age of 15 weeks. Bars represent cytokine expression standardized to *Gapdh* expression A: *Icam1*; B: *Ngal*; C: *Il6*; D: *Tnfα*; E: *Il1β*. SQ: standardized quantity. Bars represent mean ± SEM. Statistical analysis by two-way ANOVA and post-hoc Bonferroni. * = P < 0.05; ns = non-significant; sample size n = 3–5 per group (dots represent individual animals). (JPG 741 KB)

Supplemental Fig. 4. Enrichment of microglial inflammatory response genes in wild-type mice after LPS-injection at the age of 15 weeks. A: Volcano plots depicting LPS-associated DEGs in ad libitum fed and CR mice.; B: Venn diagrams depicting DEGs (logFC > 1 and adjusted-p < 0.05) from ad libitum fed WT mice injected with PBS or LPS and CR WT mice injected with PBS or LPS; sample size n = 3–5 per group (dots represent individual animals). (JPG 29 KB)

## Data Availability

Data is available upon reasonable request to the corresponding author.
