# Supplementary figures and images for: Calorie restriction increases the sensitivity of progeroid Ercc1Δ/− mice to acute (neuro)inflammation
Source: GeroScience. 2024 Sep 17;47(2):1641–52. doi: 10.1007/s11357-024-01347-1 (PMC11978592; doi:10.1007/s11357-024-01347-1)

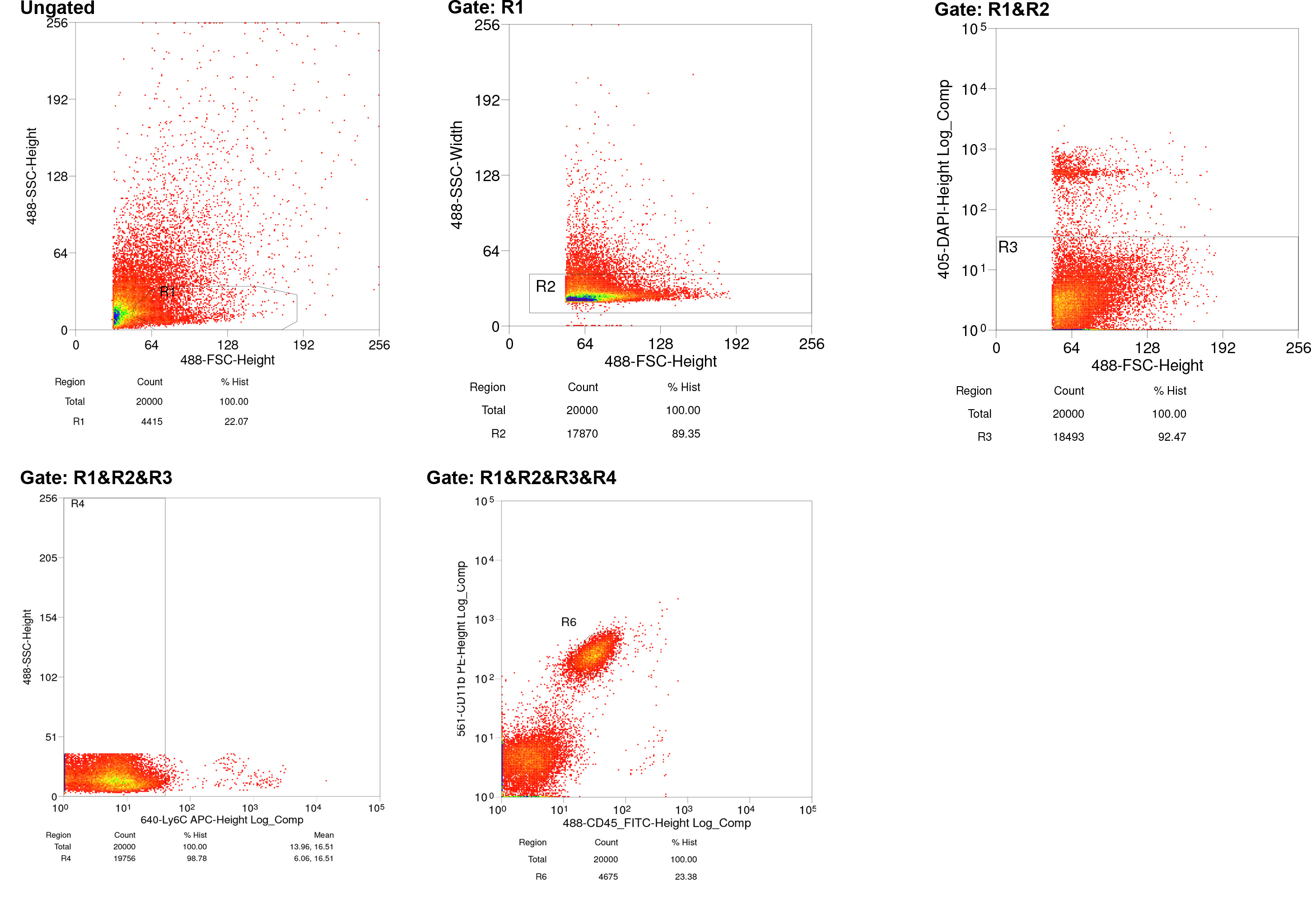

Supplement: Supplementary file 1 — Supplemental Fig. 1. Representative FACS plots. Isolated microglia were incubated with phycoerythrin (PE)-coupled rat anti-mouse CD11b (Clone M1/70, eBioscience), FITC-coupled rat anti-mouse CD45 (Clone 30-F11, eBioscience), APC-coupled rat anti-mouse Ly6c (Clone HK 1.4, Biolegend). In order to identify single cells, forward and side scatter parameters were used, and live cells were selected via the exclusion of DAPI-negative cells. Microglia were sorted by gating CD11bpos/CD45int/Ly6cneg/DAPIneg cells on a Beckman Coulter MoFlo Astrios or XDP. The gating strategy included R1 and R2 to sort cells, R3 for DAPIneg cells, R6 for CD11bpos/CD45pos, and R4 for Ly6cneg cells. (JPG 660 KB) [file 11357_2024_1347_MOESM1_ESM.jpg]

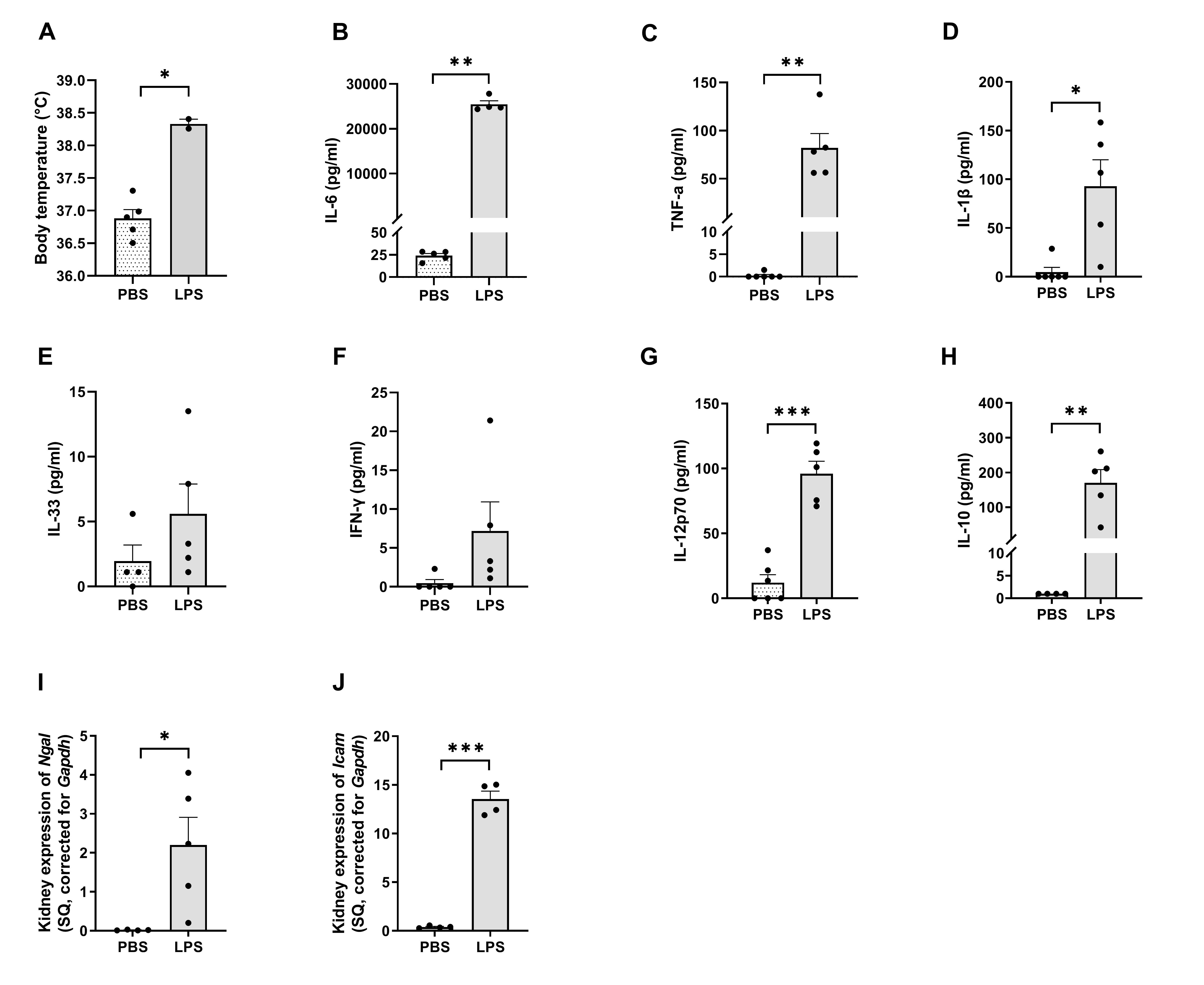

Supplement: Supplementary file 2 — Supplemental Fig. 2. LPS-injection induces a peripheral inflammatory response in wild-type mice. Effect of injection of PBS or LPS in WT mice fed ad libitum at the age of 15 weeks. A: Body temperature before and 3 h after injection; B-H: Serum cytokines.; I: Kidney expression levels of Intercellular Adhesion Molecule 1 (Icam1) corrected for Gapdh; J: Kidney expression levels of Neutrophil gelatinase-associated lipocalin (Ngal)corrected for Gapdh. Bars represent mean ± SEM. Statistical analysis by two-sided T-test. * = P < 0.05; ** = P < 0.01; *** = < 0.001; ns = non-significant; sample size n = 4–6 per group (except for body temperature, dots represent individual animals). (JPG 766 KB) [file 11357_2024_1347_MOESM2_ESM.jpg]

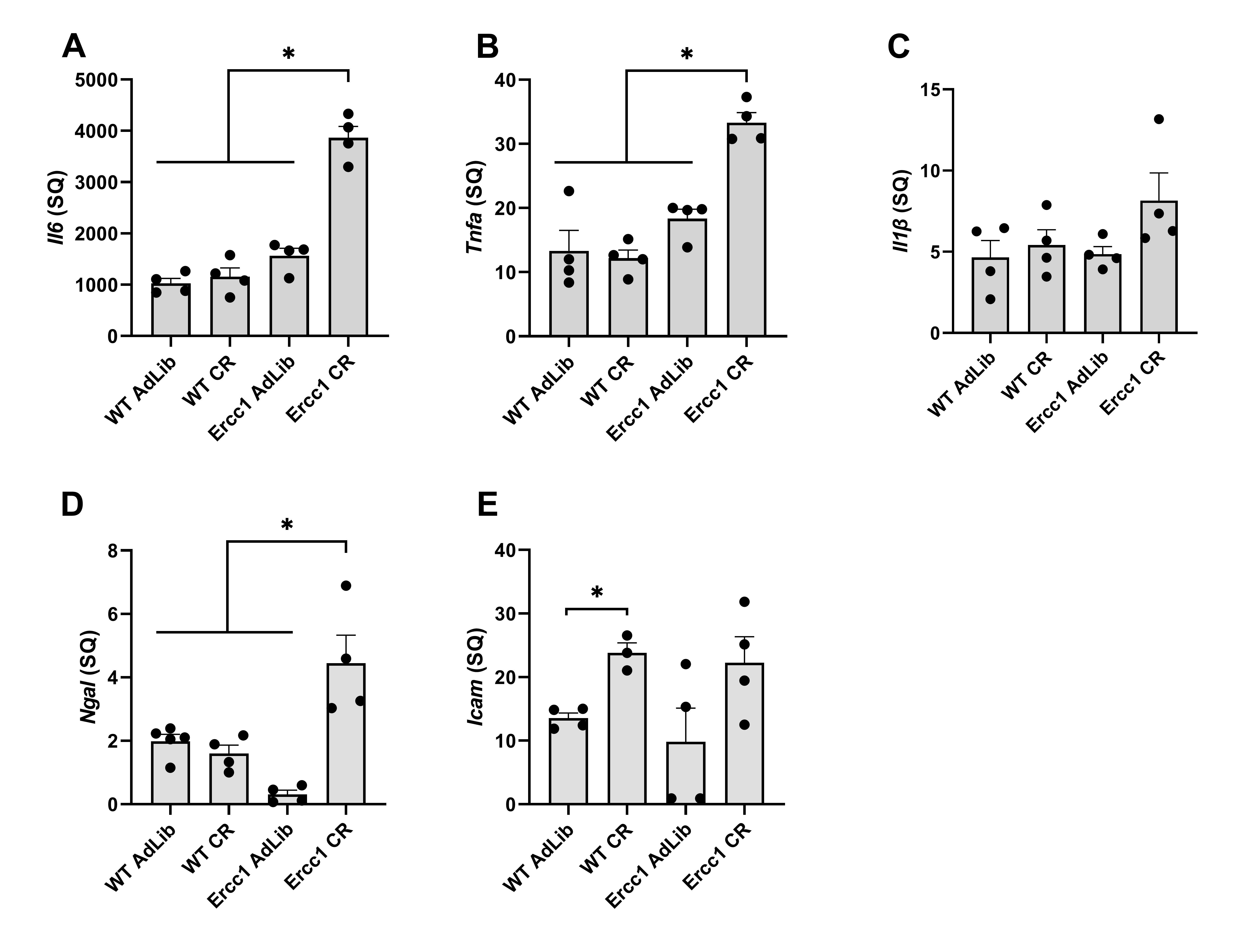

Supplement: Supplementary file 3 — Supplemental Fig. 3. LPS treatment in combination with calorie restriction increases kidney inflammatory markers in ErcclΔ/− mice versus wild-type mice. Kidney mRNA expression of Icam1, Ngal, Il6, Tnfα and Il1β after LPS-injection at the age of 15 weeks. Bars represent cytokine expression standardized to Gapdh expression A: Icam1; B: Ngal; C: Il6; D: Tnfα; E: Il1β. SQ: standardized quantity. Bars represent mean ± SEM. Statistical analysis by two-way ANOVA and post-hoc Bonferroni. * = P < 0.05; ns = non-significant; sample size n = 3–5 per group (dots represent individual animals). (JPG 741 KB) [file 11357_2024_1347_MOESM3_ESM.jpg]

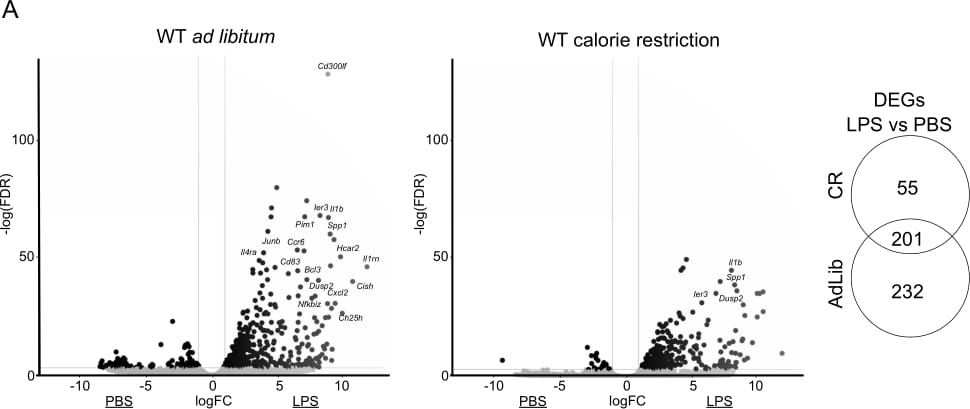

Supplement: Supplementary file 4 — Supplemental Fig. 4. Enrichment of microglial inflammatory response genes in wild-type mice after LPS-injection at the age of 15 weeks. A: Volcano plots depicting LPS-associated DEGs in ad libitum fed and CR mice.; B: Venn diagrams depicting DEGs (logFC > 1 and adjusted-p < 0.05) from ad libitum fed WT mice injected with PBS or LPS and CR WT mice injected with PBS or LPS; sample size n = 3–5 per group (dots represent individual animals). (JPG 29 KB) [file 11357_2024_1347_MOESM4_ESM.jpg]
